# PI3K/AKT signaling pathway as a critical regulator of Cisplatin response in tumor cells

**DOI:** 10.32604/or.2022.025323

**Published:** 2022-08-31

**Authors:** ZAHRA NASRPOUR NAVAEI, GHAZALEH KHALILI-TANHA, AMIR SADRA ZANGOUEI, MOHAMMAD REZA ABBASZADEGAN, MEYSAM MOGHBELI

**Affiliations:** 1 Department of Medical Genetics and Molecular Medicine, School of Medicine, Mashhad University of Medical Sciences, Mashhad, Iran; 2 Student Research Committee, Faculty of Medicine, Mashhad University of Medical Sciences, Mashhad, Iran; 3 Medical Genetics Research Center, Mashhad University of Medical Sciences, Mashhad, Iran

**Keywords:** PI3K/AKT, Cancer, Chemoresistance, Cisplatin, Chemotherapy

## Abstract

Chemotherapy is one of the main therapeutic modalities for cancer patients. Cisplatin (CDDP), as one of the first-line drugs, is of great importance in the chemotherapy of various tumors. However, a significant percentage of cancer patients are resistant to CDDP treatment. Due to the CDDP side effects on normal tissues, the diagnosis of CDDP resistance is required to suggest the most efficient therapeutic strategies for cancer patients. Several molecular mechanisms and signaling pathways are associated with CDDP response. The PI3K/AKT signaling pathway has a pivotal role in the transmission of extracellular signals into the cells to regulate various pathophysiological processes such as cell proliferation, migration, and drug resistance. In the present review, we summarized all of the studies which have been reported on the role of PI3K/AKT pathway in regulation of CDDP response. It was shown that the PI3K/AKT pathway is mainly involved in CDDP response in lung, ovarian, and gastrointestinal cancers. It was also observed that the non-coding RNAs have a key role in CDDP response by regulation of PI3K/AKT pathway. This review paves the way for suggesting a PI3K/AKT-related panel marker for the prediction of CDDP response in different cancer patients.

## Introduction

**Figure 1:**
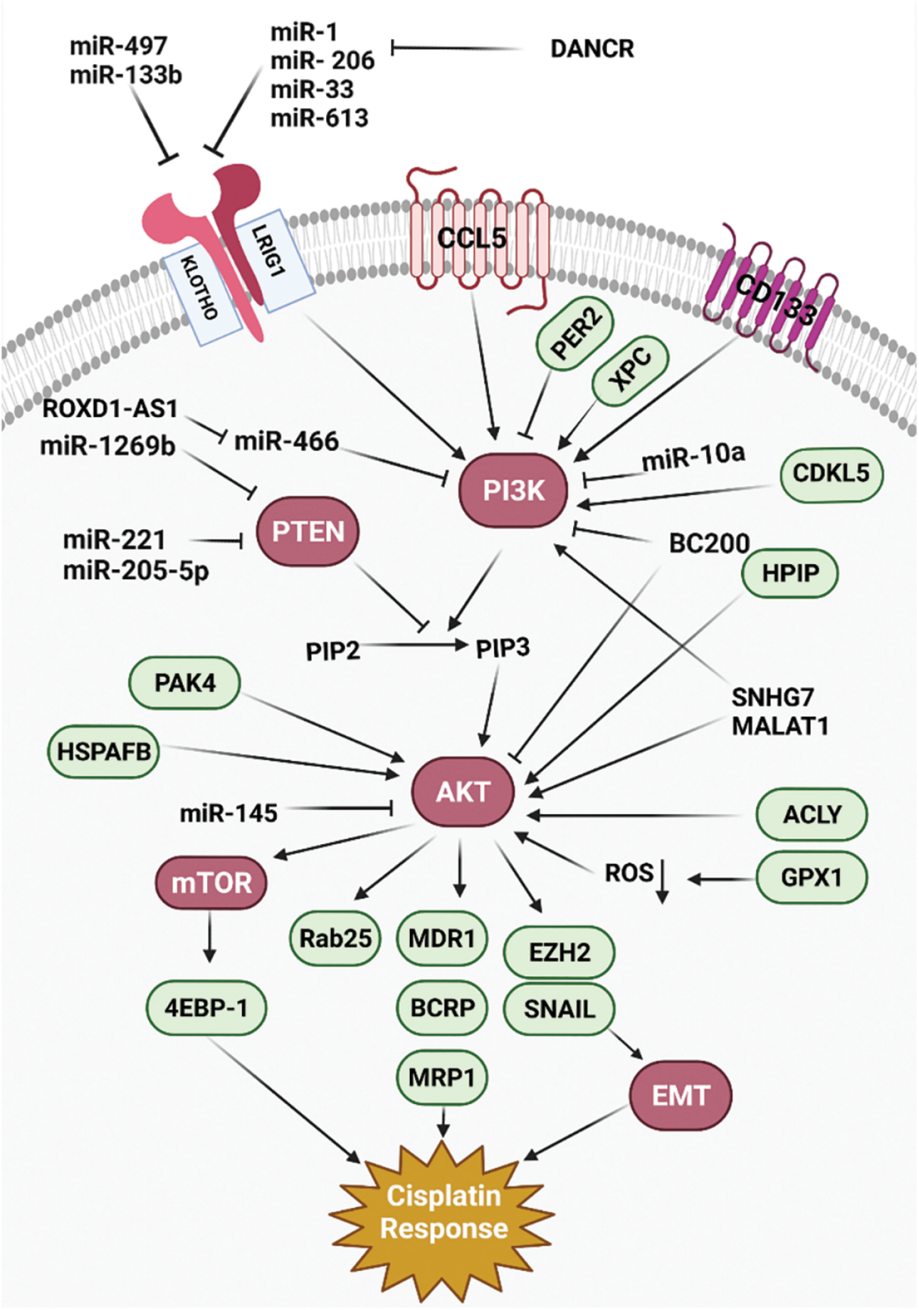
All of the molecular mechanisms that affect the Cisplatin response via PI3K/AKT signaling pathway (Created with BioRender.com).

Cisplatin (CDDP) is a common first-line chemotherapeutic drug for different cancers [1]. However, there are some limitations to the application of cisplatin, such as drug resistance and side effects. Therefore, combination therapy can reduce the CDDP drug resistance and side effects [2]. Cisplatin binds with purine bases in DNA to interfere with DNA repair and replication result in tumor cell apoptosis [3]. CDDP also promotes the reactive oxygen species (ROS) production that induces cell death through apoptosis and necrosis. Cisplatin disturbs calcium homeostasis that affects the function of mitochondrial enzymes to promote cell death [2]. Although Cancer patients are initially CDDP responsive, CDDP resistance and tumor relapse are eventually observed in the majority of patients [4]. Various molecular and cellular mechanisms are involved in CDDP resistance, including increased drug efflux, increased DNA repair, and upregulation of anti-apoptotic factors [5]. Therefore, it is required to clarify the molecular mechanisms of cisplatin resistance to improve the clinical outcome in cancer patients. PI3K/AKT signaling pathway is frequently deregulated in different cancers [6]. PI3K can be activated by tyrosine kinase receptors, and G-protein coupled receptors to produce PIP3 that activates AKT. AKT also activates various effectors such as the Mammalian target of rapamycin (mTOR) and GSK3 to regulate cell metabolism, proliferation, and motility [7]. The mTOR as a serine/threonine kinase is considered the best-characterized AKT substrate, which can be activated by phosphorylation. Subsequently, mTOR activates ribosomal protein S6 kinases (S6K) and suppresses 4E-BP, that results in elevated protein translation [8]. PI3K/AKT/mTOR pathway is involved in the sensitivity of tumor cells toward cisplatin [9,10]. It has been reported that inhibition of AKT/mTOR promoted CDDP-induced apoptosis in resistant cells [11]. AKT promotes CDDP resistance via negative regulation of P53 [12]. P13K inhibitor also increases CDDP sensitivity by Bax upregulation and cytC release [13]. PI3K/AKT activates GSK-3β that promotes β-catenin transfer to the nucleus to upregulate target genes associated with multi-drug resistance (MDR) [14]. ABC transporters such as ABCB1, ABCC1, and ABCG2 can also be upregulated by PI3K/AKT pathway to promote CDDP efflux and resistance [15,16]. PI3K/AKT pathway regulates aerobic glycolysis to prepare energy and increase the efficiency of ABC transporters for drug efflux [17]. PI3K/AKT is a promising therapeutic target for clinical tumor therapy. mTOR is one of the main effectors in PI3K/AKT pathway that regulates various cellular processes. Rapamycin analogs such as everolimus and ridaforolimus are the mTOR inhibitors that are widely used as anti-cancer agents [18]. In the present review, we have summarized all of the studies that have reported on the role of PI3K/AKT pathway in the regulation of CDDP response in tumor cells (Fig. 1). It was observed that there is a complex network of non-coding RNAs (Table 1) and protein-protein interactions (Table 2) which are responsible for CDDP response through the regulation of PI3K/AKT signaling pathway.

**Table 1:** All of the non-coding RNAs that affect Cisplatin response using the regulation of PI3K/AKT pathway

Gene	Mechanism	Target gene	Effect on the tumor cells	Clinical application	Samples	Study	Year
miR-181	Downregulation	PTEN	Increased CDDP resistance	Diagnosis	6 patients A549 cell line	Liu *et al.* [19]	2018
miR-1269b	Upregulation	PTEN	Increased CDDP resistance	Diagnosis	32 patients HA549, SPCA1, H1299 H358, PC9, A549 cell lines	Yang *et al.* [20]	2020
miR-10a	Upregulation	PIK3CA	Increased CDDP resistance	Diagnosis and prognosis	6 patients A549, H1299 cell lines	Huang *et al.* [21]	2020
miR-133b	Upregulation	EGFR	Increased CDDP sensitivity	Diagnosis	24 patients A549 cell line	Li *et al.* [22]	2018
miR-221/222	Downregulation	PTEN	Increased CDDP sensitivity	Diagnosis	A2780 cell line	Amini-Farsani *et al.* [23]	2018
miR-654-3p	Upregulation	PI3K/AKT	Increased CDDP sensitivity	Diagnosis	20 patients IGROV-1, 293T cell lines	Niu *et al.* [24]	2020
miR-21	Upregulation	PTEN	Increased CDDP resistance	Diagnosis	SGC7901 cell line	Yang *et al.* [25]	2013
miR-95-3p	Upregulation	EMP1	Increased CDDP resistance	Diagnosis and prognosis	92 patients SGC7901, AGS cell lines	Ni *et al.* [26]	2021
HOTAIR	Downregulation Upregulation	miR-34a	Increased CDDP sensitivity	Diagnosis and prognosis	27 patients SGC7901, MGC803 cell lines	Cheng *et al.* [27]	2018
FOXD1-AS1	Upregulation	miR-466	Increased CDDP resistance	Diagnosis	BGC-823, MKN28, MGC803, MKN45, AGS cell lines	Wu *et al.* [28]	2021
miR-4295	Upregulation	LRIG1	Increased CDDP resistance	Diagnosis	MKN-28, NCI-N87, SGC-7901, MKN-45, BGC-823 cell lines	Yan *et al.* [29]	2018
OIP5-AS1	Upregulation	miR-340-5p	Increased CDDP resistance	Diagnosis	MG63-CR and SAOS2-CR cell lines	Song *et al.* [30]	2019
miR-497	Upregulation	VEGFA	Increased CDDP sensitivity	Diagnosis	74 patients SAOS-2 cell line	Shao *et al.* [31]	2015
miR-221	Downregulation	PTEN	Increased CDDP sensitivity	Diagnosis	60 patients SOSP-9607, SOSP-9901, MG63, SAOS-2, U20S, hFOB1.19 cell lines	Zhao *et al.* [32]	2013
miR-145	Upregulation	AKT3	Increased CDDP sensitivity	Diagnosis and prognosis	30 patients EC109, EC9706, KYSE-150, KYSE-30, TE1 cell lines	Zheng *et al.* [33]	2019
MALAT1	Downregulation	BRWD1	Increased CDDP sensitivity	Diagnosis	Hela and C-33A cell lines	Wang *et al.* [34]	2018
miR-205-5p	Downregulation	PTEN	Increased CDDP sensitivity	Diagnosis	MG63, SAOS2 cell lines	Zhang *et al.* [35]	2019
DANCR	Upregulation	miR-33b5p, miR-33a-5p, miR-206, miR-1-3p, and miR-613	Increased CDDP resistance	Diagnosis	U87MG, U251MG, LN18, U138MG cell lines	Ma *et al.* [36]	2018

**Table 2:** All of the proteins that affect Cisplatin response using the regulation of PI3K/AKT pathway

Gene	Mechanism	Effect on the tumor cells	Clinical application	Samples	Study	Year
BC200	Upregulation	Increased CDDP resistance	Diagnosis and prognosis	76 patients H1299, A549, SKMES1, PC9, H358, SPCA1 cell lines	Gao *et al.* [37]	2019
Survivin	Upregulation	Increased CDDP resistance	Diagnosis	SW2, H82, U1285, A549, MPM cell lines	Belyanskaya *et al.* [38]	2005
Klotho	Upregulation	Increased CDDP sensitivity	Diagnosis	A549, H460 cell lines	Wang *et al.* [39]	2013
XPC	Downregulation	Increased CDDP sensitivity	Diagnosis	A549 cell line	Teng *et al.* [40]	2019
KLF5	Downregulation	Increased CDDP sensitivity	Diagnosis	H1299, A549 cell lines	Gong *et al.* [41]	2018
PAX6	Upregulation	Increased CDDP resistance	Diagnosis and prognosis	92 patients A549, SPC-A-1 cell lines	Wu *et al.* [42]	2019
GPX1	Upregulation	Increased CDDP resistance	Diagnosis and prognosis	A549, H1975, H460, H1650 cell lines	Chen *et al.* [43]	2019
USP17	Upregulation	Increased CDDP resistance	Diagnosis	A549, H1299 cell lines	Zhang *et al.* [44]	2020
HSPA12B	Upregulation	Increased CDDP resistance	Diagnosis	A549 cell line	Chen *et al.* [45]	2018
HPIP	Upregulation	Increased CDDP resistance	Diagnosis and prognosis	OAW42, PA-1, SKOV3 cell lines	Bugide *et al.* [46]	2017
HOXB4	Downregulation	Increased CDDP sensitivity	Diagnosis and prognosis	MKN-28, NCI-N87, SGC-7901, MKN-45, BGC-823 cell lines	Li *et al.* [47]	2018
Rab25	Upregulation	Increased CDDP resistance	Diagnosis	SKOV-3, ES-2 cell lines	Fan *et al.* [48]	2015
CCL5	Upregulation	Increased CDDP resistance	Diagnosis and prognosis	62 patients SKOV3 cell line	Zhou *et al.* [49]	2016
PER2	Upregulation	Increased CDDP sensitivity	Diagnosis and prognosis	SKOV3 cell line	Wang *et al.* [50]	2020
ACLY	Downregulation	Increased CDDP sensitivity	Diagnosis and prognosis	47 patients A2780, SKOV3, HEY cell lines	Wei *et al.* [51]	2021
CD133	Downregulation	Increased CDDP sensitivity	Diagnosis	KATO-111 Cell line	Lu *et al.* [52]	2019
Neogenin-1	Upregulation	Increased CDDP resistance	Diagnosis	MKN-28, BGC-823, MGC-803, SGC-7901, MKN-45 cell lines	Qu *et al.* [53]	2018
PAK4	Downregulation	Increased CDDP sensitivity	Diagnosis	49 patients AGS, MKN-45 cell lines	Fu *et al.* [54]	2014
HtrA1	Downregulation	Increased CDDP resistance	Diagnosis	SW480 cell line	Xiong *et al.* [55]	2017
MNAT1	Upregulation	Increased CDDP resistance	Diagnosis and prognosis	78 Patients MG63, U2OS, Well5, 143B cell lines	Qiu *et al.* [56]	2020
XPD	Upregulation	Increased CDDP sensitivity	Diagnosis and prognosis	20 patients EC9706, EC109 cell lines	Jian *et al.* [57]	2020
ALC1	Downregulation	Increased CDDP sensitivity	Diagnosis	TE1, TE13, ECA109, and EC9706 cell lines	Li *et al.* [58]	2019
RACK1	Upregulation	Increased CDDP resistance	Diagnosis and prognosis	Eca109, EC9706 cell lines	Liu *et al.* [59]	2018
PAK4	Upregulation	Increased CDDP resistance	Diagnosis and prognosis	93 patients Hela, CaSki cell lines	Shu *et al.* [60]	2015
PRRX1	Upregulation	Increased CDDP resistance	Diagnosis and prognosis	MCF-7 cell line	Luo *et al.* [61]	2020
Ghrelin	Upregulation	Increased CDDP resistance	Diagnosis	MDA-MB-231 cell line	Zhang *et al.* [62]	2020
SPP1	Downregulation	Increased CDDP sensitivity	Diagnosis	16 patients C-33A (Hela and CaSki) cell line	Chen *et al.* [63]	2019
Par-4	Downregulation	Increased CDDP resistance	Diagnosis	BXPC-3 cell line	Tan *et al.* [64]	2014
mTOR	Upregulation	Increased CDDP sensitivity	Diagnosis	SW1990 cell line	Li *et al.* [65]	2019
CDKL5	Upregulation	Increased CDDP resistance	Diagnosis	27 patients U87, U251 cell lines	Jiang *et al.* [66]	2020
SDC1	Upregulation	Increased CDDP resistance	Diagnosis and prognosis	30 patients HepG2 cell line	Yu *et al.* [67]	2020
HMGN5	Downregulation	Increased CDDP sensitivity	Diagnosis	UBC5637, UM-UC-3, T24 cell lines	Gan *et al.* [68]	2017
Derlin-1	Downregulation	Increased CDDP sensitivity	Diagnosis and prognosis	150 patients SV-HUC-1, BIU-87, J82, 5637 cell lines	Dong *et al.* [69]	2017
TDRG1	Upregulation	Increased CDDP resistance	Diagnosis	35 patients TCam-2 cell line	Gan *et al.* [70]	2016

## Lung Cancer

PI3K/AKT/mTOR pathway has pivotal role in tumor progression, drug resistance, and poor prognosis in various cancers [71]. PTEN also induces autophagosome formation by PI3K/AKT inhibition [72]. MicroRNAs (miRNAs) are a class of non-coding RNAs that have pivotal roles in post-transcriptional regulation by mRNA degradation or translational inhibition [73,74]. There was *miR-181* downregulation in CDDP -resistant Non-small-cell lung carcinoma (NSCLC) patients compared with controls. MiR-181 reduced cell growth and invasion, while promoting autophagy in lung tumor cells via PTEN/PI3K/AKT/mTOR axis [19]. *MiR-1269b* induced cell proliferation and CDDP resistance by *PTEN* targeting that resulted in PI3K/AKT signaling activation in lung tumor cells. There was also significant *miR-1269b* up-regulation in NSCLC patients that was correlated with CDDP resistance and survival [20]. PIK3CA as a subunit of PI3K has a pivotal role in PI3K/AKT/mTOR activation. There was *miR-10a* upregulation in circulating lung tumor cells that were associated with poor prognosis by induction of CDDP resistance. *MiR-10a* promoted CDDP resistance through *PIK3CA* targeting in circulating lung tumor cells [21]. There was a direct association between the levels of *BC200* expression and clinicopathological features of the tumor, including tumor stage and lymph node involvement in NSCLC patients. *BC200* knockdown significantly downregulated the PI3K, AKT, and STAT3. It induced NSCLC metastasis and regulated the cisplatin-induced apoptosis via PI3K/AKT pathway [37]. *MiR-133b* reduced lung tumor cell proliferation, while promoting apoptosis by CCND1 downregulation and Bax upregulation, respectively. It also downregulated the EGFR, p-STAT3, and p-JAK2 in cisplatin-induced lung tumor cells. Therefore, *miR-133b* reduced cell proliferation by EGFR targeting that mediated PI3K/AKT in cisplatin-induced NSCLC cells [22].

Apoptosis consists of two converging cascades, including extrinsic and intrinsic pathways [75]. Death ligands are initiators of the extrinsic pathway by a death-inducing signaling complex that results in caspase-8 activation. However, the intrinsic pathway is associated with the release of cytochrome c from mitochondria that activates caspases. Inhibitors of apoptosis proteins (IAP) are endogenous caspases inhibitors in intrinsic and extrinsic pathways [76]. Survivin belongs to the IAPs that regulate cell proliferation and suppress apoptosis [77]. AKT has a pivotal role in regulation of PI3K signaling, which is implicated in cell proliferation and metabolism [78]. It has been shown that cisplatin resistance can be associated with cisplatin-induced AKT activation that results in survivin induction in SCLC. There was survivin upregulation in SCLC cells following the cisplatin treatment [38].

Klotho is a transmembrane protein that functions as an inhibitor of the IGF-1 pathway [79]. PI3K/AKT is also a downstream cascade of IGF-1 pathway with pivotal roles in regulation of apoptosis and CDDP response [80,81]. There was significant klotho downregulation and p-AKT upregulation in CDDP-resistant lung tumor cells. Klotho upregulation significantly increased the Bax/Bcl-2 ratio in CDDP resistant cells. Therefore, klotho alleviated the CDDP resistance by regulation of PI3K/AKT pathway and Bax/Bcl-2 expression ratio in lung tumor cells [39].

Nucleotide excision repair is involved in the repair of DNA damages caused by CDDP [82]. Xeroderma pigmentosum complementation group C (XPC) is a key detector of DNA damage [83]. It has been reported that XPC silencing significantly reduced cell proliferation while promoting apoptosis in A549/CDDP cells. XPC silencing also significantly increased the Bax/Bcl-2 ratios in A549/CDDP cells, while downregulated the p-AKT. Therefore, XPC inhibition significantly induced apoptosis in CDDP-resistant lung tumor cells [40].

ATP-binding cassette proteins (ABC) are trans-membrane proteins involved in multi-drug resistance [84]. BCRP belongs to the ABC protein family that pumps intracellular drug to reduce intracellular concentration and increase drug resistance [85]. Long noncoding RNAs (lncRNAs) are a class of none-coding RNAs involved in regulation of miRNAs as competing endogenous RNAs (ceRNAs) [86]. Deregulation of lncRNAs is involved in cell proliferation, apoptosis, migration, and drug resistance [87]. *SNHG7* knockdown increased cisplatin-sensitivity by downregulations of MRD1 and BCRP in NSCLC. There was also SNHG7 upregulation in NSCLC tissues compared with normal margins which was correlated with advanced-stage and CDDP-resistant. Since *SNHG7* silencing significantly downregulated the PI3K, p-AKT, and p-mTOR in cisplatin-resistant NSCLC cells, *SNHG7* promoted cisplatin-resistance via MRD1 and BCRP upregulations through PI3K/AKT pathway in NSCLC [88].

Hypoxia is commonly observed in solid tumors that affects pathophysiological processes such as angiogenesis and drug resistance [89]. Hypoxia inducible factor-1α (HIF-1α) is a pivotal regulator of cell proliferation and glycolysis during hypoxia response [90]. Krüppel-like factor 5 (KLF5) is a developmental transcription factor that regulates the levels of HIF-1α expression [91]. There was a significant KLF5 upregulation in hypoxic NSCLC cells. *KLF5* knockdown reduced the levels of P-gp and HIF-1α expressions in hypoxic NSCLC cells. *KLF5* knockdown also suppressed HIF-1α-dependent glycolysis through PI3K/AKT/mTOR inactivation that resulted in reduced hypoxia-induced cisplatin resistance [41].

Epithelial-to-mesenchymal transition (EMT) is a pathophysiological process in which the tumor cells lose their epithelial properties to obtain the mesenchymal phenotype [92,93]. Following the EMT, mesenchymal tumor cells obtain invasive and metastatic features [94]. It has been shown that there was *PAX6* upregulation in NSCLC, which was significantly associated with lower overall survival (OS). PAX6 was involved in regulation of ZEB2 as a critical factor in EMT-mediated self renewal, thereby promoting cisplatin resistance in NSCLC. PAX6-ZEB2 also induced tumor invasion via PI3K/AKT dependent downregulation of CDH1 in NSCLC [42]. *AFAP1-AS1* is an oncogenic lncRNA that encodes AFAP1 gene antisense [95]. There was *AFAP1-AS1* upregulation in CDDP-resistant NSCLC tissues and cells. *AFAP1-AS1* silencing reduced cell proliferation and migration via regulation of EMT process and PI3K/AKT pathway. It significantly promoted EMT in CDDP-resistant NSCLC cells. *AFAP1-AS1* also inhibited apoptosis by regulation of EZH2 to activate PI3K/AKT pathway which increased CDDP resistance in NSCLC cells [96].

ROS is normally produced during cellular metabolism that regulates cell proliferation and apoptosis. Apoptosis-related ROS is a fundamental mechanism of CDDP [97]. ROS is produced by mitochondria due to the cisplatin treatment [98]. Glutathione peroxidase 1 (GPX1) is an antioxidant enzyme involved in ROS metabolizing. It has been reported that there was GPX1 upregulation in NSCLC cells. GPX1 inhibited cisplatin-induced ROS accumulation that promoted PI3K-AKT pathway. GPX1 downregulation promoted apoptosis via increased ROS accumulation and AKT suppression in CDDP-resistant NSCLC cells [43].

Deubiquitinating enzymes (DUBs) are the inhibitors of protein degradation by removing the ubiquitin chains [99]. Ubiquitin-specific protease 17 (USP17) belongs to the DUB protein family that regulates cell migration, inflammation, and tumor progression. USP17 regulates the Ras pathway to affect cell migration and proliferation [100,101]. It has been reported that CDDP increased the levels of *USP17* expression. USP17 also induced the NSCLC cell proliferation through PI3K/AKT activation [44]. HSPA12B belongs to the HSP70 protein family that is directly associated with CDDP resistance through p-IκBα and p-AKT upregulations and caspase-3 downregulation in lung tumor cells [45].

## Ovarian Cancer

Cisplatin resistance can be developed by increased drug efflux, increased detoxification, induced DNA repair, and reduced drug-induced apoptosis [102]. Cancer stem cells (CSCs) and EMT process are contributed with the chemoresistance and tumor relapse in ovarian cancer patients [103]. PI3K/AKT/mTOR signaling has a pivotal role in regulation of cell cycle, cell proliferation, and chemoresistance [104]. There was E-cadherin downregulation, while N-cadherin and Vimentin upregulations in cisplatin resistant ovarian tumor cells compared with sensitive cells. EMT process was along with increased levels of CSC markers in CDDP-resistant ovarian tumor cells. Suppression of PI3K/AKT/mTOR axis significantly inhibited the EMT and CSC features [105]. The hematopoietic PBX interaction protein (HPIP) is an oncogene that activates PI3K/AKT, MAPK, and SHH signaling pathways to promote tumor progression and metastasis [106–108]. *HPIP* silencing inhibited AKT and MAPK in ovarian tumor cells. HPIP promoted cisplatin resistance of ovarian cancer cells. There was a direct association between the levels of HPIP expression and higher tumor grades. HPIP activated the PI3K/AKT pathway, which resulted in E-cadherin downregulation, while stabilized Snail in ovarian tumor cells [46]. The Homeobox (HOX) family of transcription factors are pivotal regulators of developmental processes and tumor progressions [109]. There was *HOXB4* upregulation in CDDP-resistant ovarian cancer cells. Silencing of *HOXB4* increased CDDP sensitivity by PI3K/AKT suppression that resulted in ABC transporters downregulations in ovrian tumor cells [47].

Deregulation of miRNAs is one of the main reasons of chemotherapeutic resistance by targeting the drug response genes in various cancers [110]. As a tumor suppressor, PTEN is involved in regulation of cell growth, migration, and apoptosis [111]. It has been reported that there was a significant miR-221/222 upregulation in ovarian tumor cells. MiR-221/222 downregulation promoted apoptosis and cisplatin sensitivity through PTEN targeting and subsequent PI3K/AKT activation in ovarian cancer [23]. There was significant *miR-654-3p* downregulation in CDDP-resistant ovarian tumor cells. *MiR-654-3p* suppressed cell proliferation and migration while promoting the CDDP sensitivity by inactivation of PI3K/AKT pathway. It also suppressed P-gp via QPRT targeting which may associate with CDDP sensitivity in ovarian tumor cells [24]. Rab25 belongs to the GTPase protein family that has key roles in cell proliferation, signal transduction, and cytoskeletal organization [112]. PI3K/AKT signaling upregulated the Rab25 to induce CDDP resistance in ovarian tumor cells [48].

Autophagy is associated with drug response via different signaling pathways in tumor cells [113,114]. STAT3 has a dual function in regulation of autophagy in which the nuclear STAT3 positively regulates the transcription of autophagy-related genes, while cytoplasmic STAT3 has a negative role by interacting with FOXO1 and FOXO3 transcription factors. The mitochondrial STAT3 also inhibits the oxidative stress that is promoted by autophagy and maintains the mitochondria from mitophagy-related degradation [115]. It has been reported that STAT3 induced EMT while inhibited the autophagy that resulted in tumor cell proliferation, invasion, and cisplatin resistance through Slug and MAPK in ovarian cancer. STAT3 also suppressed the autophagy that resulted in cisplatin resistance following the activation of the PI3K/AKT and MEK/ERK pathways in ovarian tumor cells [116].

Cancer-associated fibroblasts (CAFs) as the main stromal cells in the tumor microenvironment can also be affected by the cisplatin treatment that can interfere with the normal tissue homeostasis. CCL5 belongs to the CC-chemokine family that has pivotal roles in drug resistance and metastasis. STAT3 is a transcriptional modulator of chemokines responses and growth factors that regulates cell invasion and chemotherapy resistance [117]. PI3K/AKT signaling pathway can also be activated by CCL5 [118]. It has been reported that CAFs promoted CDDP resistance via CCL5 production in ovarian tumor cells. CCL5 induced CDDP-resistance through the p-AKT and p-STAT3 pathways [49]. PER2, as a circadian factor, has critical role during tumor progression by apoptosis induction [119,120]. It has been shown that PER2 increased CDDP sensitivity in ovarian tumor cells by PI3K and AKT downregulations. It also increased the levels of caspase 3 expressions that resulted in apoptosis induction. There were significant serum TNF-α and IL-6 downregulations in PER2 upregulated tumors before the CDDP treatment. Therefore, PER2 reduced systemic inflammation to promote CDDP sensitivity in ovarian cancer patients [50].

Growth hormone secretagogue receptor (GHSR) is activated by Ghrelin that has a key role in tumor progression [121]. Ghrelin is observed in acylated and unacetylated forms in blood. The acetylated gherlin is the most active while less abundant form that activates secretagogue receptor type 1a (GHS-R1a). The unacylated ghrelin is the most abundant while less active form that functions independently of GHS-R1a [122]. Acetylated gherlin promoted the cisplatin resistance and cell proliferation in ovarian tumor cells. Gherlin mediates its role through GHS-R1a and activation of PI3K/AKT pathway that results in p53 and PUMA downregulations in ovarian cancer [123]. ATP citrate lyase (ACLY) catalyzes the conversion of the citrate and coenzyme A to acetyl CoA and oxaloacetate (OAA) [124]. Acetyl-CoA has a critical role in transcriptional regulation by histones acetylation. OAA is also an important substrate for aspartate production that is required for nucleotide synthesis and redox reactions [125]. There was significant ACLY upregulation in CDDP-resistant ovarian tumor cells. *ACLY* knockdown reduced cell proliferation, while promoted apoptosis in ovarian tumor cells via *CCND1* and *CDK4* downregulations and *P16* upregulation. Knockdown of *ACLY* reduced the cell proliferation and CDDP resistance via p-AKT suppression and P16–CCND1–CDK4 axis regulation in ovarian cancer [51].

## Gastric and Colorectal Cancers

Cancer stem cells are a subpopulation of tumor cells involved in chemotherapeutic resistance and tumor relapse [126]. They obtain drug resistance by upregulation of extrusion pumps and DNA repair proteins. Therefore, conventional anti-tumor agents are not effective in the elimination of CSCs and they cause recurrence [127]. CD133 is a glycoprotein involved in activation of the PI3K/AKT pathway through PI3K-p85 [128]. It has been reported that there were *CD133* upregulations in cisplatin-resistant gastric tumor cells compared with sensitive cells. CD133 increased CDDP resistance via PI3K/AKT/mTOR activation in gastric tumor cells [52].

MiRNAs and lncRNAs have diverse functions in cellular processes such as cell differentiation, proliferation, and apoptosis. PTEN is a negative regulator of PI3K/AKT by dephosphorylation of PIP3. MiR-22-3p promoted cisplatin sensitivity though *PTEN* upregulation and PI3K/AKT inhibition in gastrointestinal tumors [129]. There was a significant *miR-21* upregulation in cisplatin resistant gastric tumor cells in comparison with sensitive cells. *MiR-21* regulated the PTEN expression as a pivotal factor in drug resistance [25]. There was a significant *miR-95-3p* upregulation in CDDP-resistant compared with sensitive gastric tumor cells. *MiR-95-3p* induced CDDP resistance, cell proliferation, and migration through EMP1 targeting that activated PI3K/AKT pathway in gastric tumor cells [26]. Knockdown of *HOTAIR* reduced cisplatin resistance and tumor growth through miR-34a upregulation that resulted in suppression of the PI3K/AKT and WNT signaling pathways in gastric tumor cells. There was also *HOTAIR* upregulation in GC tissues compared with normal margins [27]. *MALAT1* induced cisplatin resistance of GC cells. There was a significant correlation between the levels of *MALAT1* expression and poor survival in GC patients. *MALAT1* promoted GC proliferation and invasion by p-PI3K and p-AKT upregulations and activation of PI3K/AKT pathway in gastric tumor cells. *MALAT1* was also involved in CDDP resistance by regulation of Bcl-2 [130].

ZEB1 and ZEB2 are zinc finger EMT-transcription factors [94]. Neogenin-1 (Neo1) is a transmembrane receptor and member of the immunoglobulin superfamily [131]. Neo1 promoted cell migration and cisplatin resistance in gastric tumor cells. It also induced EMT process by activation of the Rac1/PI3K/AKT axis that resulted in ZEB1, CDH2, and VIM upregulations, while CDH1 downregulation in gastric tumor cells [53]. P21-activated kinases (PAKs) have critical roles in regulation of angiogenesis, EMT, and metabolic processes [132,133]. PAK4 is a key regulator of tumor cell migration that is down stream effector of Met receptor [134]. It has been reported that PAK4 promoted CDDP resistance by activation of PI3K/AKT pathways in gastric tumor cells [54]. There was significant *UCA1* upregulation in gastric tumor tissues compared with normal margins which was correlated with lymph node involvement, distant metastasis, and advanced tumor stage. *UCA1* also induced CDDP resistance through EZH2 targeting and PI3K/AKT activation in gastric tumor cells [135].

4E-BP1 is a translational inhibitor and down stream target of the PI3K/AKT/mTOR pathway [136,137]. *FOXD1-AS1* activated the PI3K/AKT/mTOR signaling via *miR-466* sponging and subsequent PIK3CA release that resulted in 4E-BP1 hyperphosphorylation. Activation of 4E-BP1 also induced eIF4E and eIF4G interaction that upregulated the FOXD1 protein to promote CDDP resistance in gastric tumor cells. Therefore, *FOXD1-AS1* increased CDDP resistance via translational regulation of FOXD1 in gastric cancer [28]. EGFR is a key signaling pathway during tumor progression that functions by various intracellular cascades, such as MAPK and PI3K/AKT to promote cell proliferation and invasion [138,139]. LRIG1 is a negative regulator of the EGFR [140]. It has been shown that *miR-4295* induced cell proliferation, while suppressed the CDDP-induced apoptosis in gastric tumor cells through LRIG1 targeting and subsequent activation of EGFR/PI3K/AKT axis [29].

HtrA1 belongs to the serine protease protein family involved in cell proliferation, apoptosis, and embryogenesis [141]. XIAP is an anti apoptotic factor that inhibits caspase-3, caspase-7, and caspase-9 [142]. HtrA1 has a critical role in XIAP targeting and degradation during the chemotherapeutic responses [143]. It has been reported that HtrA1 downregulation increased CDDP resistance through XIAP and PI3K/AKT activation in colon tumor cells [55].

## Osteosarcoma

MRP1 and P-gp belong to the ATP-binding cassette (ABC) transporters that are involved in drug resistance [144,145]. There was a significant *OIP5-AS1* upregulation in the cisplatin-resistant osteosarcoma (OS) cells compared with sensitive cells. *OIP5-AS1* silencing inhibited cell proliferation and reduced cisplatin resistance in OS cells. Reduced levels of *OIP5-AS1* expression significantly inhibited the PI3K/AKT/mTOR axis. *OIP5-AS1* regulated the cisplatin resistance through the PI3K/AKT/mTOR pathway via *miR-340-5p* targeting and subsequent LPAATβ upregulation in OS cells [30]. Cyclin-dependent kinase-activating kinase (CAK) complex contains CDK7, Cyclin H, and MNAT1 [146]. It has been shown that there were *MNAT1* upregulations in osteosarcoma tissues that were correlated with poor prognosis. *MNAT1* downregulation suppressed osteosarcoma cell proliferation, invasion, and *in-vivo* growth. It also promoted cisplatin resistance through PI3K/AKT/mTOR activation [56].

There is a close correlation between the autophagy and chemoresistance. Autophagy maintains the cellular homeostasis by removing the damaged cellular components via autophagosomes. Therefore, it maintains a balance between the synthesis and degradation to protect the cells during nutrient deprivation [147,148]. However, it can also promote apoptosis due to the excessive proteins lack [149]. It has been reported that *miR-22* regulated CDDP-resistance via autophagy inhibition in osteosarcoma cells. *MiR-22* suppressed CDDP induced autophagy and reduced drug resistance through PI3K/AKT/mTOR axis in osteosarcoma [150]. There was a significant *miR-497* downregulation in osteosarcoma tissues compared with normal margins. *MiR-497* downregulation promoted cisplatin resistance via PI3K/AKT pathway by VEGFA targeting in osteosarcoma [31]. PTEN dephosphorylates PIP3 to suppress AKT activity [151]. It has been shown that *miR-221* promoted cell proliferation and CDDP resistance, while reduced apoptosis via PTEN targeting and subsequent PI3K/AKT activation in osteosarcoma cells. *MiR-221* also regulated the levels of Bcl-2, CCND1, and p27. There was also *miR-221* upregulation in osteosarcoma tissues [32].

## Esophageal Squamous Cell Carcinoma

Tumor malignancy is associated with genome instability which is the result of aberrant DNA repair, replication, and chromosome segregation. Xeroderma pigmentosum complementation group D (XPD) is a DNA helicase involved in DNA repair that is caused by oxidative stress [152]. There was a significant XPD downregulation in esophageal squamous cell carcinoma (ESCC) tissue compared with normal margins. XPD upregulation significantly suppressed the cell proliferation and migration, while increased CDDP sensitivity by suppression of the PI3K/AKT pathway [57]. ABC transporters are among the most important proteins involved in efflux of anticancer drugs which reduce the chemotherapeutics efficacy. Multidrug resistance-associated protein 1 (MRP1) is closely associated with opposed chemotherapeutic outcomes [153]. It has been observed that *miR-145* increased CDDP sensitivity of ESCC cells through MRP1 and P-gp downregulations following the AKT3 targeting and subsequent PI3K/AKT inhibition. *MiR-145* suppressed anti-apoptotic factors including CCND1 and Bcl-2, while induced Bax through PI3K/AKT inhibition in ESCC cells [33].

Amplified in liver cancer 1 gene (ALC1) belongs to the chromatin remodeling enzymes involved in tumor cell proliferation and metastasis [154]. There was *ALC1* upregulation in ESCC cells. *ALC1* knockdown reduced cell growth and increased CDDP sensitivity in ESCC cells through inactivation of PI3K/AKT pathway and subsequent glycolysis suppression. *ALC1* knockdown also upregulated the caspase-3/7 and increased apoptosis in ESCC cells that induced CDDP sensitivity in ESCC cells [58]. RACK1 belongs to the WD40 repeat protein family that functions in protein anchoring and shuttling between cellular compartments. It is also involved in transcriptional and translational regulations [155]. As a scaffold protein, RACK1 binds with kinases and membrane-bound receptors to regulate cell proliferation, adhesion, and migration [156]. It has been observed that RACK1 increased cell proliferation and resistance toward the CDDP induced apoptosis in ESCC. RACK1 promoted CDDP-resistance by PI3K/AKT activation and subsequent Bcl-2 upregulation in ESCC cells [59].

## Cervical and Breast Cancers

PAKs are serine/threonine kinases characterized as the effectors of Rac and Cdc42 [157]. They have pivotal roles in tumor progression by regulation of the Ras-induced metabolism, cell proliferation, angiogenesis, and EMT [133,158]. PAK4 belongs to the PAKs family which is involved in cervical cancer progression and cisplatin resistance. There was a significant PAK4 upregulation in cervical tumor tissues compared with normal margins which were correlated with FIGO stage, grade, and lymph node involvement. PAK4 also promotes the cisplatin resistance in cervical tumor cells through PI3K/AKT pathway [60]. P-glycoprotein (P-gp) is encoded by ABCB1 which induces chemoresistance [159]. Paired-related homeobox 1 (PRRX1) is a transcriptional co-activator that promotes EMT process during tumor progressions [160]. It was shown that PRRX1 reduced chemosensitivity of breast tumor cells by P-gp upregulation. PRRX1 also increased PI3K and AKT phosphorylations following the PTEN suppression in breast cancer [61].

Ghrelin is a hormone produced by the stomach that is involved in energy homeostasis via regulation of adipocyte function and glucose metabolism. Ghrelin reduced the CDDP sensitivity by activation of PI3K/AKT/mTOR signaling, Bcl-2 upregulation, and caspase-3 downregulation in breast tumor cells. PI3K/AKT/mTOR axis regulated the ghrelin-induced anti-apoptosis in breast tumor cells treated with cisplatin [62]. Secreted phosphoprotein 1 (SPP1) is a cytokine and cell-matrix adherence protein associated with regulation of cell proliferation, apoptosis, and migration. It has been observed that there was SPP1 upregulation in cervical cancer tissues. SPP1 suppression reduced CDDP resistance through inhibition of PI3K/AKT pathway [63]. BRWD1 is a developmental transcriptional factor that has a pivotal role in chromatin remodeling and transcriptional regulation. It has been shown that *MALAT1* downregulation induced the cisplatin sensitivity in cervical tumor cells through regulating BRWD1 and cell apoptosis. The p-PI3K and p-AKT were also up-regulated following *MALAT1* over expression [34].

## Nasopharyngeal and Pancreatic Cancers

EMT is a cellular process that mediates differentiation of epithelial to mesenchymal cells via suppression of adhesion molecules, while upregulation of mesenchymal proteins. This process facilitates separation of tumor cells from primary tumors and invasion to the secondary sites [161]. EMT is orchestrated by E-cadherin (epithelial factor) downregulation, while Vimentin and N-cadherin (mesenchymal factors) upregulations [162]. EMT has a critical role in regulation of drug resistance [163]. PI3K/AKT signaling pathway is involved in regulation of tumor invasion and drug resistance [164]. PTEN is considered as a negative regulator of PI3K/AKT pathways in which it dephosphorylates the PIP3 which results in AKT inactivation and PI3K/AKT suppression [165]. *MiR-205-5p* promoted the ECM degradation by MMP-9 and MMP-2 up-regulations. It downregulated Ecadherin, while upregulated the Vimentin, N-cadherin, Slug, and Snail in nasopharyngeal tumor cells. Therefore, *miR-205-5p* induced EMT through PTEN inhibition in cisplatin-resistant NPC cells [35]. Prostate apoptosis response-4 (Par-4) is a pro-apoptotic factor highly expressed in apoptosis-induced prostate tumor cells [166]. It has been shown that Par-4 downregulation promoted CDDP resistance through stimulation of PI3K/AKT-dependent EMT in pancreatic tumor cells [64]. mTOR is one of the main effectors of PI3K/AKT that integrates the inputs from growth factors [167]. It is also a sensor of nutrients and oxygen [168]. It has been shown that the mTOR inhibited AKT phosphorylation to increase the cisplatin sensitivity in pancreatic tumor cells [65].

## Other Cancers

AXL belongs to the receptor tyrosine kinase (RTK) protein family that activates PI3K/AKT pathway to promote cell proliferation and regulate tumor drug resistance [169,170]. It has been reported that there was an inverse association between the levels of *DANCR* expression and CDDP sensitivity in glioma cells. *DNACR* promoted cisplatin resistance via *miR-33b-5p*, *miR-33a-5p*, *miR-206*, *miR-1-3p*, and *miR-613* sponging and AXL upregulation. *DANCR* activated the PI3K/AKT/NF-κB axis by increased AKT and IκBα phosphorylations [36]. Cigarette smoking increases urothelial tumor aggressiveness by promoting tumor proliferation and angiogenesis. Nicotine functions through different receptors, such as nicotinic acetylcholine receptors and/or EGF receptors [171]. It regulates PI3K/AKT pathway in different cancer cells [172,173]. It has been reported that nicotine promoted the CDDP resistance by PI3K/AKT/mTOR activation [174].

Cyclin-dependent kinase-like 5 (CDKL5) is a positive regulator of PI3K. There was CDKL5 upregulation in glioma tissues compared with normal samples. CDKL5 upregulation promoted CDDP drug resistance, cell migration, and proliferation in glioma cells via phosphorylation of PI3K and AKT and subsequent PI3K/AKT activation [66]. Syndecan 1 (SDC1) is a heparan sulfate proteoglycan involved in cell attachment, signaling, and cytoskeletal organization [175]. It has been reported that there was SDC1 upregulation in advanced stage and drug resistant liver tumors. There was also a correlation between AKT activation and SDC1 upregulation, which resulted in cisplatin resistance [67].

HMGN5 is a developmental transcriptional regulator and chromatin remodeler [176]. There was an inverse association between the levels of HMGN5 expressions and CDDP sensitivity in urothelial bladder tumor cells. *HMGN5* knockdown induced CDDP sensitivity through suppression of PI3K/AKT signaling and subsequent cytochrome c, caspase-3, and PARP upregulations that confirmed the activation of the intrinsic apoptosis pathway [68]. Derlin-1 is a pivotal factor involved in the elimination and the retrotranslocation of misfolded proteins from endoplasmic reticulum [177]. There was Derlin-1 upregulation in bladder tumor tissues and cells. Derlin-1 also promoted chemoresistance through activation of PI3K/AKT/Bcl-2 axis in bladder tumor cells [69]. Testis developmental related gene 1 (TDRG1) induces seminoma cell proliferation and invasion via activation of PI3K/AKT pathway [178]. It positively regulated the p-mTOR and affected the cell cycle progression in seminoma cells during CDDP treatment. TDRG1 regulated the CDDP sensitivity through the PI3K/AKT/mTOR signaling and intrinsic apoptosis pathway in seminoma cells [70].

## Conclusions

CDDP is a widely used first-line anti-cancer drug in various cancers. Since, CDDP has severe side effects on different normal organs and tissues in cancer patients; it is required to determine the CDDP-resistant from sensitive tumors. Therefore, clarification of the molecular mechanisms involved in CDDP response provides novel therapeutic strategies in chemo-resistant patients. In the present review, we summarized all of the studies that have been assessed the role of PI3K/AKT pathway in Cisplatin response. It was shown that the PI3K/AKT signaling pathway regulates the Cisplatin response via different cellular mechanisms such as apoptosis, autophagy, DNA repair, ABC transporters, and EMT process. The PI3K/AKT was mainly involved in regulation of CDDP response in lung, ovarian, and gastrointestinal tumors. It was also observed that the non-coding RNAs are the pivotal regulators of Cisplatin response via PI3K/AKT pathway. Non-coding RNAs mainly affected the PI3K/AKT pathway through the RTK and PTEN regulations. This review paves the way of suggesting a PI3K/AKT related panel marker for the prediction of Cisplatin response in cancer patients. Since PI3K/AKT mainly promotes the Cisplatin resistance in different tumors, suppression of this pathway through the RTK, PI3K, and mTOR inhibitors or specific miRNAs can be efficient methods to overcome the CDDP resistance and improve the quality of life in cancer patients. Moreover, the microRNAs that are involved in regulation of CDDP response by targeting the PI3K/AKT signaling can be introduced as the non-invasive markers for the prediction of CDDP response among cancer patients. The clinical non-invasive application of PI3K/AKT related miRNAs also reduces the CDDP side effects and paves the way to select the efficient therapeutic methods in a personalized medicine that significantly improves the quality of life and patient’s survival. A combination of PI3K/AKT inhibitors with CDDP can also be a promising therapeutic modality among the cancer patients who show the CDDP resistance through PI3K/AKT signaling pathway.
